# Urinary Thrombin: A Novel Marker of Glomerular Inflammation for the Diagnosis of Crescentic Glomerulonephritis (Prospective Observational Study)

**DOI:** 10.1371/journal.pone.0118704

**Published:** 2015-03-05

**Authors:** Yasunori Kitamoto, Kenji Arizono, Hiroyoshi Fukui, Kimio Tomita, Hiroshi Kitamura, Yoshio Taguma, Takahisa Imamura

**Affiliations:** 1 Department of Laboratory Medicine, JCHO Sendai Hospital, Sendai, Japan; 2 Department of Nephrology, Kumamoto Chuo Hospital, Kumamoto, Japan; 3 Department of Nephrology, Faculty of Life Sciences, Kumamoto University, Kumamoto, Japan; 4 Department of Nephrology, JCHO Sendai Hospital, Sendai, Japan; 5 Department of Molecular Pathology, Faculty of Life Sciences, Kumamoto University, Kumamoto, Japan; University of Louisville, UNITED STATES

## Abstract

**Background:**

Crescentic glomerulonephritis (CresGN), an uncommon rapidly progressive disease, is characterized by severe glomerular inflammation with fibrin deposition. The lack of specific CresGN biomarkers delays diagnosis and threatens life. Because fibrin deposits in CresGN glomeruli indicate thrombin generation, we hypothesized that thrombin is excreted in urine and is a specific CresGN biomarker.

**Methods:**

We measured urinary thrombin activity in 200 untreated patients (17 with CresGN, 183 with primary glomerulonephritis) and controls (8 patients with healed CresGN, 11 with nephrosclerosis, and 10 with tubulointerstitial nephritis, and 66 healthy volunteers). CresGN types included 15 pauci-immune and 2 immune complex. We assessed the diagnostic accuracy of thrombinuria in 169 patients with hematuria and proteinuria. Renal biopsy tissues were immunostained for tissue factor and fibrin. We analyzed the relationship of thrombinuria to plasma thrombin-antithrombin complex, hematuria, proteinuria, glomerular filtration rate, glomerular fibrin deposition, antineutrophil cytoplasmic antibodies (ANCAs), and C-reactive protein (CRP). We studied changes in thrombin activities after glucocorticoid treatment in 12 patients with thrombinuria.

**Results:**

The highest thrombinuria occurrence was in CresGN (70.6%), followed by membranoproliferative glomerulonephritis (41.7%), IgA nephropathy (9.2%), and acute glomerulonephritis (0%). More than 75% of patients with nonproliferative glomerulonephritis manifested no thrombinuria. No controls had thrombinuria. Thrombinuria showed high CresGN specificity (90.1%) and moderate sensitivity (70.6%) and was detected in 4 of 7 patients with ANCA-negative CresGN. In CresGN, thrombinuria was associated with fibrin deposition in glomerular extracapillary tissue, where monocytes/macrophages expressed tissue factor. Thrombinuria in CresGN was unrelated to plasma thrombin-antithrombin complex, hematuria, proteinuria, glomerular filtration rate, and CRP. After glucocorticoid treatment, thrombinuria in patients with CresGN rapidly disappeared but proteinuria and hematuria persisted.

**Conclusions:**

Thrombinuria was specific for glomerular inflammation, was unaffected by systemic inflammation or coagulation, and demonstrated good diagnostic accuracy for CresGN including ANCA-negative cases. Thrombinuria measurement may provide risk-free diagnosis and screening for CresGN.

## Introduction

Crescentic glomerulonephritis (CresGN), an uncommon [1] but devastating disease, rapidly progresses to renal failure [2]; however, patients receiving proper treatment at early stages may heal without impaired renal function. Therefore, early diagnosis and treatment are crucial for improving the poor prognosis of this disease. Since Bright’s report in 1827 [3,4], proteinuria, caused by an impaired barrier against plasma protein leakage at the glomerular capillary wall [5], has been a marker for glomerular diseases caused by inflammation, hypertension, and metabolic or hereditary disorders, but proteinuria is not specific for CresGN. Serum antineutrophil cytoplasmic antibodies (ANCAs) are used to diagnose pauci-immune CresGN, but a significant proportion (10–30%) of patients with pauci-immune CresGN [6] and most patients with immune-complex CresGN are negative for ANCAs [7]. Thus, a substantial number of patients with CresGN remain undiagnosed if only the ANCA test is used. Definitive diagnosis of CresGN requires histopathological examination of renal biopsy tissues. However, biopsy is invasive, causes patients pain, and risks serious bleeding, and routine sections from biopsy tissues do not always include the characteristic crescentic lesions, which are focally or segmentally distributed. Thus, noninvasive and specific diagnostic methods have been sought for early CresGN diagnosis.

Inflammation triggers the tissue factor pathway of blood coagulation [8]. Tissue factor is expressed in extravascular inflamed tissues, where plasma leaks because of increased vascular permeability [9], and activates blood coagulation factors (inactive precursors) in the leaked plasma. The ultimate product, thrombin, converts fibrinogen to fibrin, which deposits in lesions. CresGN features severe glomerular inflammation and glomerular crescent formation, often with fibrin deposition in glomerular extracapillary tissue [10] that is caused by thrombin probably generated via the monocyte/macrophage tissue factor-dependent coagulation pathway [11–15]. The pathogenesis of experimental CresGN [16] involved thrombin and stimulation of its receptor, protease-activated receptor-1 [17]. Thrombin may also contribute to glomerular inflammation by modulating monocyte/macrophage chemotaxis [18]. These close associations of thrombin with CresGN suggest that this protease may be a CresGN biomarker. However, thrombin has not been investigated in patients with CresGN, probably because no assay method exists for lesional thrombin activity.

Urine reflects the glomerular milieu and is obtained without pain or risk, so urine may be an appropriate material for estimating thrombin generation in inflamed glomeruli. Therefore, to investigate the feasibility of using urinary thrombin as a diagnostic indicator of CresGN, we utilized a urinary thrombin assay [19,20] to evaluate patients with various glomerulonephritides, which led us to propose the application of urinary thrombin activity to CresGN diagnosis and screening.

## Methods

### Patients and sample collection

We studied 200 patients with untreated glomerulonephritis (17 patients with CresGN and 183 patients with other types of primary glomerulonephritis) who were admitted to Sendai Shakaihoken Hospital or Kumamoto Chuo Hospital from 2003 to 2011 (Table 1). In our 17 patients with CresGN (11 from Kumamoto Chuo Hospital, 6 from Sendai Shakaihoken Hospital), more than 50% of glomeruli had crescentic lesions in renal biopsy specimens (diffuse CresGN); 15 patients had the pauci-immune type of the disease and 2 had the immune-complex type (1 IgA nephropathy [IgAN], 1 rheumatoid arthritis). Patients with healed CresGN (N = 8) were age- and sex-matched with patients who had untreated CresGN. We judged CresGN healed when the abnormal urinary findings (proteinuria and hematuria) disappeared. For ANCA-associated CresGN, we added the disappearance of serum ANCAs to judge healing. Nephrosclerosis (N = 11) and tubulointerstitial nephritis (TIN; N = 10) were studied as a representative non-inflammatory glomerular disease and as a representative tubulointerstitial inflammatory disease, respectively. Our study also included healthy volunteers (N = 66) who visited our hospital for a routine health check and had no abnormal urinary findings or impaired renal function and were receiving no anticoagulant, antiplatelet, or anti-inflammatory drugs. Patients with primary glomerulonephritis included 87 patients with IgAN, 9 with non-IgA mesangial proliferative glomerulonephritis (non-IgA mesPGN), 12 with membranoproliferative glomerulonephritis (MPGN), 9 with acute glomerulonephritis (AGN), 13 with minimal change glomerulopathy (MCNS), 17 with focal segmental glomerulosclerosis (FSGS), and 36 with membranous glomerulopathy (MN).

**Table 1:** Characteristics of the patients.

Disease	No. of patients (%)	Sex (male/female)	Age (yr) (mean±SD)*	Proteinuria (g/gCr) (mean±SD)†	eGFR (ml/min/1.73 m^2^) (mean±SD)‡
Crescentic glomerulonephritis (CresGN)	17 (8.5)	9/8	64.1±12.7	2.79±2.58	14.7±9.5
IgA nephropathy (IgAN)	87 (43.5)	35/52	32.6±14.4	1.09±2.22	81.1±30.7
Non-IgA mesangial proliferative glomerulonephritis (non-IgA mesPGN)	9 (4.5)	3/6	46.7±17.3	0.75±1.37	72.6±24.1
Membranoproliferative glomerulonephritis (MPGN)	12 (6.0)	7/5	39.9±15.5	2.21±2.08	64.7±21.5
Acute glomerulonephritis (AGN)	9 (4.5)	6/3	41.9±20.7	1.37±1.87	68.7±24.2
Minimal change glomerulopathy (MCNS)	13 (6.5)	8/5	41.6±18.9	6.04±2.43	83.4±20.0
Focal segmental glomerulosclerosis (FSGS)	17 (8.5)	6/11	46.1±14.8	4.05±3.80	67.9±34.7
Membranous glomerulopathy (MN)	36 (18.0)	21/15	62.9±10.8	4.99±6.76	74.0±24.2
Subtotal	200 (100)				
Healed crescentic glomerulonephritis	8	4/4	66.9 ± 10.7	Negative§	40.8 ± 22.8
Nephrosclerosis	11	4/7	61.0 ± 9.7	0.63±0.90	54.2 ± 29.9
Tubulointerstitial nephritis (TIN)	10	5/5	50.8. ± 19.6	0.54±0.56	27,9 ± 17.2
Healthy volunteers	66	42/24	41.4±10.3	Negative§	82.9±15.7

*The ages of patients with healed CresGN, CresGN, MN, or nephrosclerosis were significantly higher than those of the other groups (*P*<0.01) except for the patients with TIN. The ages of patients with healed CresGN and CresGN were significantly higher than those of patients with with TIN at *P*<0.05. The age of patients with IgAN was significantly lower than that of patients with non-IgA mesPGN, FSGS, or TIN and that of healthy volunteers at *P*<0.01 and was significantly lower than that of patients with MPGN and MCNS at *P*<0.05.

†Patients with MCNS and MN had significantly heavier proteinuria compared with patients with IgAN, non-IgA mesPGN, AGN, nephrosclerosis, or TIN at *P*<0.01 and patients with MPGN or CresGN at *P*<0.05. Patients with FSGS manifested heavier proteinuria than patients with IgAN at *P*<0.01 and patients with non-IgA mesPGN, nephrosclerosis, or TIN at *P*<0.05. gCr indicates gram of urinary creatinine.

‡For the estimated glomerular filtration rate (eGFR), patients with CresGN had a significantly lower eGFR than did patients with healed CresGN (*P*<0.05) and the other patients (*P*<0.01) except those with TIN. Patients with TIN had a significantly lower eGFR than did the other groups except patients with CresGN or healed CresGN. Patients with healed CresGN had a significanty lower eGFR than the other patients except those with CresGN, nephrosclerosis, or TIN. Healthy volunteers had significantly higher eGFR values than the other groups except patients with IgAN, non-IgA mesPGN, AGN, MCNS, or MN. Patients with FSGS and MPGN had significantly lower values than did healthy volunteers, and patients with FSGS had a significantly lower value than did patients with IgAN at *P*<0.05.

§Negative proteinuria results were obtained via the test tape method.

Ethics Committees of Sendai Shakaihoken Hospital (approval number: 2004003) and Kumamoto Chuo Hospital (approval number: H15003) approved this study, and all patients and volunteers gave written informed consent for participation.

We used clinical examination findings, including pathological studies of renal biopsy specimens, to diagnose all consecutive patients, after which they received standard treatment. We diagnosed AGN by evaluating a combination of clinical features and abnormal serological findings including hypocomplementemia and an elevated titer of antistreptolysin O (7 patients) or human parvovirus B19-specific IgM antibodies [21] (2 patients). We collected blood and urine samples within 24 hours before renal biopsy except for patients with AGN, whose samples we collected when the patients manifested hypocomplementemia. Urine samples obtained at the second morning void within 2 hours after the first void were centrifuged at 370 × *g* for 10 minutes at 4°C; supernatants were promptly stored at -80°C until use.

### Thrombin measurement

We measured urinary thrombin activity by using the cleaving activity of *t*-butyloxycarbonyl-l-valyl-l-prolyl-l-arginine 4-methyl-coumaryl-7-amide (Peptide Research Institute, Minoh, Japan), which was inhibited by hirudin, a thrombin-specific inhibitor, as reported previously [19]. Briefly, we measured the hydrolytic activity of the substrate in urine with a fluorescence spectrophotometer (excitation at 380 nm, emission at 440 nm), which was connected to a recorder, for 5 minutes or longer to obtain the linear fluorescence increase. We then added a molar excess of hirudin and measured the fluorescence for 5 minutes. We determined the urinary thrombin activity as hirudin-inhibited cleavage activity, expressed as thrombin units calibrated by using 1 mU human thrombin (Calbiochem, San Diego, CA) as a standard. To investigate the influence of urinary retention in the bladder on thrombin activity, we incubated 9 thrombin-positive urine samples without additives for 2 hours at 37°C and then compared those thrombin activities with activities before incubation.

### Diagnostic accuracy of urinary thrombin for CresGN

Among 200 patients, 169 patients (17 with CresGN, 82 with IgAN, 9 with non-IgA mesPGN, 12 with MPGN, 9 with AGN, 4 with MCNS, 9 with FSGS, and 27 with MN) presented with hematuria and proteinuria as determined via the test tape method. Using the urine samples of these patients, we evaluated the diagnostic accuracy of thrombinuria in patients with CresGN via receiver operating characteristic (ROC) curves.

### Histological studies

We counted the number of crescentic glomeruli in periodic acid-Schiff-stained CresGN biopsy tissue sections that were 2 μm thick and determined the percentage of crescentic glomeruli or cellular crescentic glomeruli vs. the total number of glomeruli. Deparaffinized 2-μm-thick biopsy tissue sections were immunostained with anti-tissue factor monoclonal antibody [22] (MoAb) and an APAAP (alkaline phosphatase anti-alkaline phosphatase) kit (Dako Japan, Tokyo, Japan) according to the manufacturer’s instructions and were then counterstained with Mayer’s hematoxylin. Normal mouse IgG served as the control for the MoAb. For fluorescence immunostaining, deparaffinized tissue sections from patients with CresGN were treated with anti-tissue factor mouse MoAb, followed by biotin-labeled anti-mouse IgG (SeraCare Life Sciences, Gaithersburg, MD) and rhodamine-labeled streptavidin (Rockland Immunochemicals, Gilbertsville, PA) in that order. After sections were incubated in excess mouse IgG and biotin for 30 min at room temperature and were washed, they were treated with a complex of anti-mouse macrophage MoAb (KP-1; Dako Japan) and fluorescein isothiocyanate-labeled anti-mouse IgG goat IgG (0.2 μg/ml; BD Biosciences Pharmingen, San Jose, CA) for 30 min at room temperature. We then studied the sections with a fluorescence microscope. Fibrin deposits in fresh-frozen sections (4 μm thick) were stained with fluorescein isothiocyanate-labeled rabbit antibody against human fibrinogen (Medical and Biological Laboratories, Nagoya, Japan).

### Measurement of plasma and urinary thrombin-antithrombin complex (TAT), urinary hemoglobin, and serum ANCAs

We used an enzyme-linked immunosorbent assay to measure plasma TAT [23] in 48 patients (9 patients [16 measurements] with ANCA-associated CresGN, 39 patients with primary glomerulonephritis) for the estimation of thrombin released into the circulation. We also measured TAT levels in the urine of 20 patients with glomerulonephritis. We quantified hematuria (glomerular bleeding) in 46 patients with glomerulonephritis by measuring urinary hemoglobin levels [24]. We used an enzyme-linked immunosorbent assay to measure serum myeloperoxidase-ANCA [25] and proteinase 3-ANCA [26] in patients with CresGN.

### Effect of glucocorticoids on thrombinuria

We measured thrombin activities in 12 thrombinuria-positive patients (6 with CresGN, 3 with IgAN, and 3 with relapsed MCNS) before and after 2 weeks of glucocorticoid treatment. We did not measure urinary thrombin activities in the rest of the patients because sample volumes were insufficient for proper analysis.

### Statistical analysis

We used Fisher’s protected least significant difference test in StatView 7 from Abacus Concepts (Berkeley, CA) to compare characteristics of patients, to compare thrombin excretion levels in patients with glomerulonephritides, and to compare estimated glomerular filtration rate (eGFR) [27] and proteinuria values in patients with and without thrombinuria. Correlations and associations were tested via regression analysis and Fisher’s z-transformation, respectively. The stability of urinary thrombin activity and changes in thrombinuria and proteinuria levels after steroid treatment were analyzed by using the non-parametric test (Wilcoxon signed-rank test). ROC curve analysis was performed by using MedCalc (version 9.3.1.0). A *P* value of <0.05 indicated statistical significance.

## Results

### Thrombinuria in patients and controls

**Fig 1:**
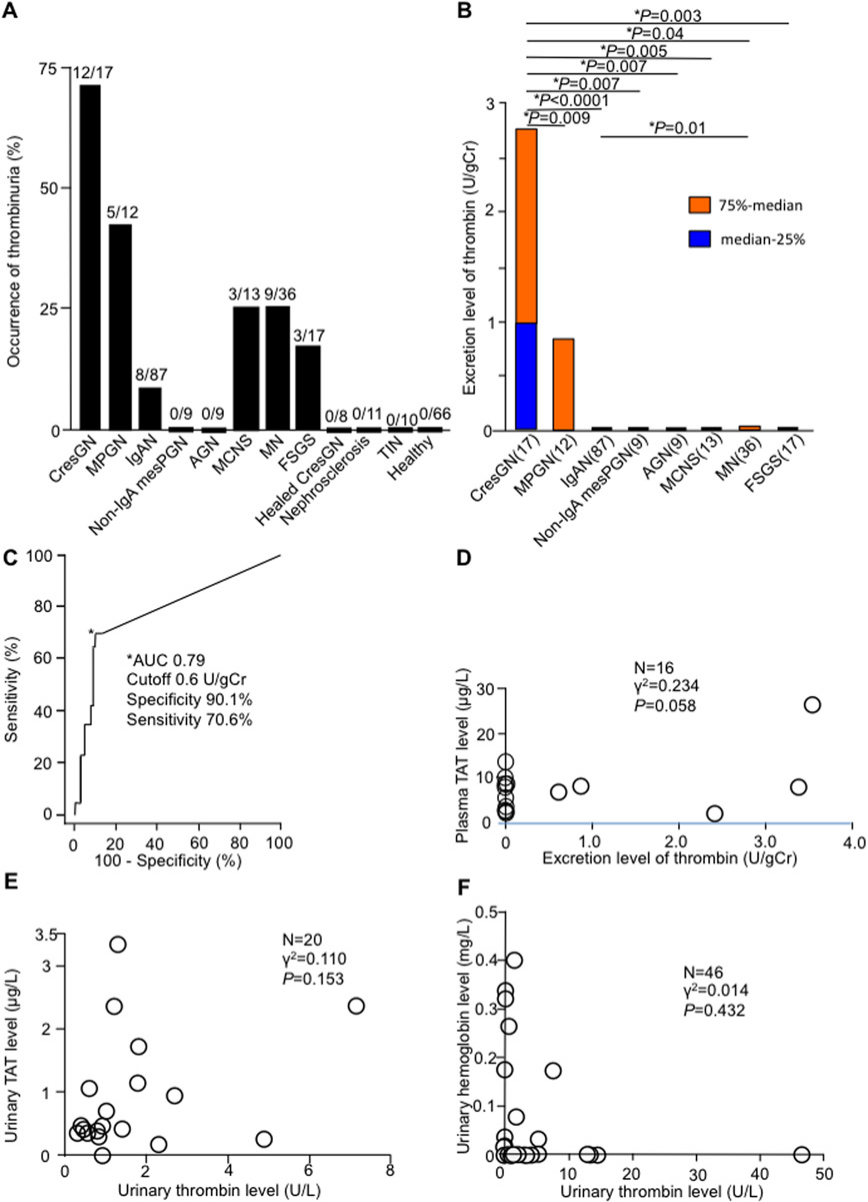
Thrombinuria in glomerulonephritis and its diagnostic accuracy for crescentic glomerulonephritis (CresGN) and its relation to plasma thrombin-antithrombin complex (TAT), urinary TAT, and hemoglobinuria. (**A**) Occurrence of urinary thrombin detected (>0.2 U/L) in each group; fractions over the columns indicate the number of samples with thrombin activity per the total number of samples. (**B**) Thrombin excretion level for each type of glomerulonephritis, as shown by box plots that indicate the median-25th and 75th-median percentiles. Bars with asterisks (*) show only significant differences between two groups. MPGN, membranoproliferative glomerulonephritis; IgAN, IgA nephropathy; non-IgA mesPGN, non-IgA mesangial proliferative glomerulonephritis; AGN, acute glomerulonephritis; MCNS, minimal change glomerulopathy; MN, membranous nephropathy; FSGS, focal segmental glomerulosclerosis; and TIN, tubulointerstitial nephritis. gCr indicates gram of urinary creatinine. (**C**) The receiver operating characteristic (ROC) curve for diagnosis of CresGN in patients with hematuria and proteinuria. The asterisk indicates the diagnostic accuracy at the cutoff value of 0.6 U/gCr. CI indicates confidence interval. (**D**) Relation of thrombin excretion to plasma TAT. Circles indicate patients with ANCA-associated CresGN (16 measurements in 9 patients). gCr indicates gram of creatinine. (**E**) Relation of urinary thrombin to urinary TAT. (**F**) Relation of urinary thrombin to hemoglobinuria. In (**E**) and (**F**), each circle represents an individual patient with glomerulonephritis.

With regard to patients with proliferative glomerulonephritis, 70.6% of patients with CresGN, 41.7% of patients with MPGN, 9.2% of patients with IgAN, and no patients with non-IgA mesPGN or AGN manifested thrombinuria (Fig. 1A). With regard to patients with nonproliferative glomerulonephritis, 23.1% of patients with MCNS, 25.0% of patients with MN, and 17.6% of patients with FSGS demonstrated thrombinuria. None of the controls (healed CresGN, nephrosclerosis, patients with TIN, and healthy volunteers) evidenced thrombinuria. CresGN patients had the highest average thrombin excretion level (Fig. 1B). From ROC curve analysis (Fig. 1C), the area under the curve was 0.79, and the specificity and sensitivity were 90.1% and 70.6%, respectively, at a cutoff value of 0.6 U/g of creatinine.

### Stability of urinary thrombin activity

Urinary thrombin activities after a 2-hour incubation at 37°C varied from 77.1% to 100% (average: 89.4% ± 8.7%) of initial values, which means that the decrease in urinary thrombin activity (*P*<0.01) because of urinary retention in the bladder was too slight to affect the reliability of the urinary thrombin assays.

### Relation between thrombinuria and plasma or urinary TAT and between thrombinuria and urinary hemoglobin levels

Plasma TAT levels showed no significant association with thrombin excretion levels in patients with ANCA-associated CresGN (Fig. 1D), in which thrombin is supposedly generated by injured vascular endothelial cells and released into circulation, and showed no association in patients with primary glomerulonephritis (*P* = 0.899, γ^2^ = 0.0004, N = 39). Urinary TAT levels were also not associated with urinary thrombin levels in patients with glomerulonephritis (Fig. 1E), nor were urinary hemoglobin levels associated with urinary thrombin levels in other patients with glomerulonephritis (Fig. 1F).

### Relation between thrombinuria and ANCAs, CRP, and crescent formation in CresGN

In 17 patients with CresGN, 10 with the pauci-immune type of CresGN were positive for myeloperoxidase-ANCA but the other 5 with the pauci-immune type of CresGN and 2 with the immune-complex type of CresGN were negative for myeloperoxidase-ANCA (Table 2). All patients were negative for proteinase 3-ANCA and anti-glomerular basement membrane antibody. Four of the 7 ANCA-negative patients with CresGN and 2 of the 5 ANCA-negative patients with pauci-immune CresGN presented with thrombinuria. Two of 5 thrombinuria-negative patients with CresGN (all had pauci-immune disease) were positive for myeloperoxidase-ANCA. Three patients with CresGN (all with pauci-immune disease) were negative for both ANCA and thrombin. Therefore, 14 of 17 (82.4%) patients with CresGN and 12 of 15 (80%) patients with pauci-immune CresGN were positive for either thrombinuria or myeloperoxidase-ANCA. Serum CRP levels were not associated with thrombinuria in CresGN (*P* = 0.526, γ^2^ = 0.027, N = 17) (Table 2). No significant association was found between thrombinuria and percentages of total crescent formation (*P* = 0.242, γ^2^ = 0.090, N = 17) or cellular crescent formation (*P* = 0.152, γ^2^ = 0.132, N = 17).

**Table 2:** Characteristics of patients with crescentic glomerulonephritis.

Case*	Age/sex	Immune complex deposition in glomeruli	Antineutrophil cytoplasmic antibodies	Glomeruli with crescents (%)	Glomeruli with cellular crescents (%)	eGFR† (ml/min/1.73 m^2^)	Proteinuria (g/gCr)†	Thrombin activity (U/gCr)†	C-reactive protein (mg/dl)
1s	78/M	Pauci-immune	Myeloperoxidase	50	14	11	4.09	0.86	1.61
2s‡	69/F	Immune complex	Negative	50	0	24.6	1.46	1.00	4.31
3s	61/F	Pauci-immune	Myeloperoxidase	94	56	8.1	4.65	61.49	0.09
4k	64/F	Pauci-immune	Negative	63	6	16.7	1.17	0.95	0.86
5k	72/M	Pauci-immune	Myeloperoxidase	75	0	15.9	1.77	3.37	4.16
6k	59/M	Pauci-immune	Myeloperoxidase	67	67	8	1.34	2.13	13.29
7s‡	27/M	Immune complex	Negative	56	6	17.1	3.86	3.35	0.36
8s	66/M	Pauci-immune	Myeloperoxidase	100	43	15.1	2.79	1.38	2.5
9k	73/F	Pauci-immune	Myeloperoxidase	86	0	4.9	0.39	3.03	0.86
10k	55/F	Pauci-immune	Negative	50	33	27.1	2.04	0.97	0.05
11s	69/M	Pauci-immune	Myeloperoxidase	67	0	41.1	0.42	0.7	8.03
12k	75/F	Pauci-immune	Myeloperoxidase	100	0	3.4	10.63	2.76	12.1
13k	71/M	Pauci-immune	Myeloperoxidase	60	0	13.2	1.56	0**	1.19
14k	47/M	Pauci-immune	Negative	91	0	6.7	0.92	0**	2.33
15k	76/M	Pauci-immune	Negative	57	29	14.2	6.2	0**	0.52
16k	70/F	Pauci-immune	Negative	70	70	17.9	1.24	0**	1.27
17k	57/F	Pauci-immune	Myeloperoxidase	100	0	5.1	2.82	0**	0.47

*Cases from Sendai Shakaihoken Hospital and Kumamoto Chuo Hospital are shown by s and k, respectively.

†eGFR indicates estimated glomerular filtration rate; gCr, gram of urinary creatinine.

‡Cases 2 and 7 were associated with rheumatoid arthritis and IgA nephropathy, respectively.

**Activities less than 0.6 U/gCr were regarded as zero.

### Tissue factor expression in glomeruli

**Fig 2:**
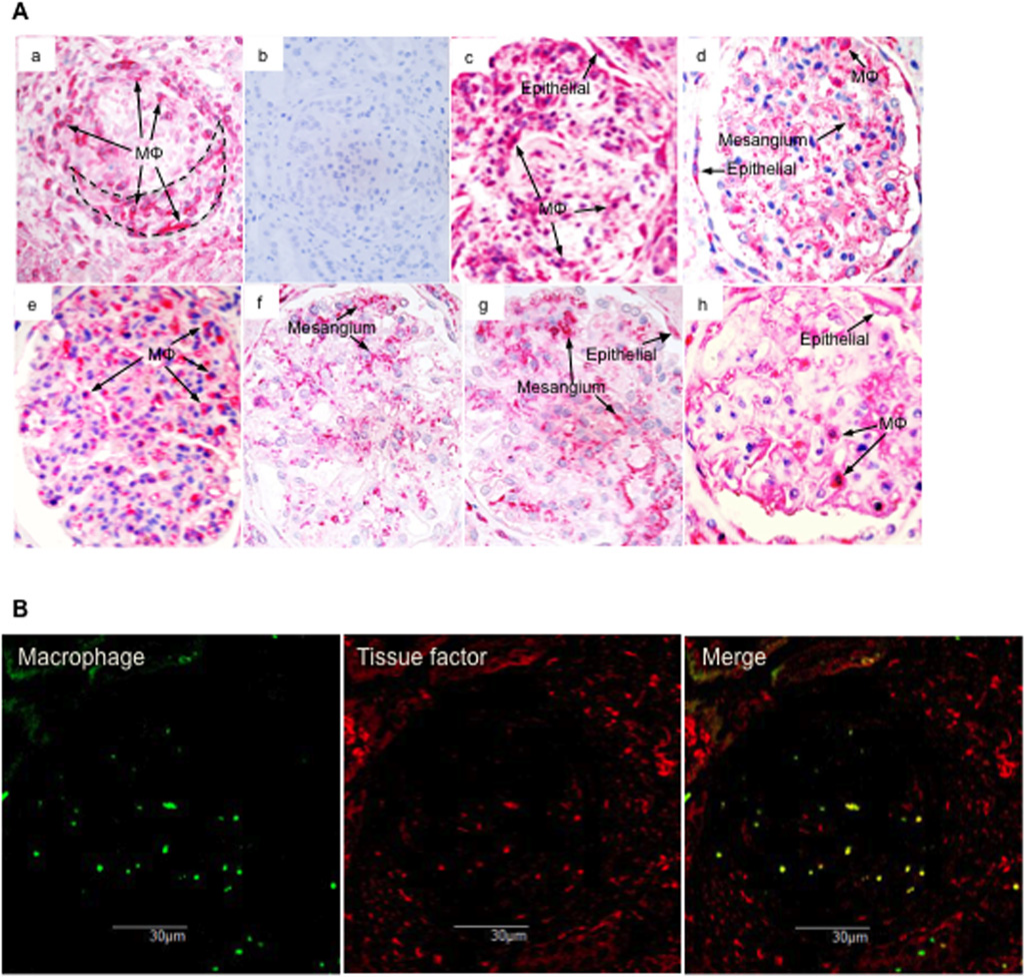
Glomerular tissue factor expression in biopsy tissues. (**A**) Representative tissue factor expression patterns of renal biopsy tissues as shown by immunostaining. All patients, except those with acute glomerulonephritis (AGN), had thrombinuria. (**a**) Crescentic glomerulonephritis (CresGN); (**b**) control staining of CresGN with normal mouse IgG; (**c**) membranoproliferative glomerulonephritis; (**d**) IgA nephropathy; (**e**) AGN; (**f**) minimal change glomerulopathy; (**g**) focal segmental glomerulosclerosis; and (**h**) membranous nephropathy. Mφ indicates monocytes/macrophages; Epithelial, epithelial cells; and Mesangium, mesangial areas. The broken line in (**a**) outlines a cellular crescent. (**B**) Double-staining pattern of CD68 (macrophages) and tissue factor in the CresGN glomerulus.

Monocytes/macrophages in glomerular (extracapillary) tissue of patients with CresGN expressed tissue factor (Fig. 2). Monocytes/macrophages in glomerular (extracapillary) tissue of patients with MPGN, IgAN, or MN also expressed tissue factor, as did monocytes/macrophages in glomerular capillaries of patients with AGN; glomerular epithelial cells in patients with MPGN, IgAN, FSGS, or MN; and mesangial areas in patients with IgAN, MCNS, or FSGS (Fig. 2A).

### Relation between thrombinuria and glomerular fibrin deposition

Glomerular fibrin is used clinically as a histological marker of activated coagulation in glomeruli. About one-half of patients with CresGN had double-positive results for thrombinuria and glomerular fibrin deposition and about one-third of patients had single-positive results, but only one-fifth of patients evidenced double-negative findings (Table 3). For other glomerulonephritides, patients with double-positive results were rare, and no patients with IgAN, non-IgA mesPGN, or MCNS had double-positive findings. Glomerular fibrin deposition occurred in 28% and 38% of patients with IgAN and non-IgA mesPGN, respectively, but patients with MCNS demonstrated no glomerular fibrin deposition.

**Table 3:** Thrombinuria and fibrin deposition in glomerular tissues in glomerulonephritis.

Type of glomerulonephritis (no. of patients)	Thr(+)/Fib(+)*	Thr(+)/Fib(-)	Thr(-)/Fib(+)	Thr(-)/Fib(-)
CresGN (15)	7	3	2	3
IgAN (83)	0	8	23	52
Non-IgA mesPGN (8)	0	0	3	5
MPGN (10)	1	3	3	3
MCNS (8)	0	3	0	5
FSGS (13)	2	1	2	8
MN (27)	2	3	7	15

*Thr and Fib indicate thrombinuria and fibrin deposition, respectively. Fibrin deposition was determined via immunofluorescence microscopy, and thrombin activities > and below the detection limit (0.2 U/L) were regarded as positive (+) and negative (-) thrombinuria, respectively.

CresGN indicates crescentic glomerulonephritis; IgAN, IgA nephropathy; Non-IgA mesPGN, non-IgA mesangial proliferative glomerulonephritis; MPGN, membranoproliferative glomerulonephritis; MCNS, minimal change glomerulopathy; FSGS, focal segmental glomerulosclerosis; and MN, membranous glomerulopathy.

### Glucocorticoid treatment effects on thrombinuria and proteinuria

**Fig 3:**
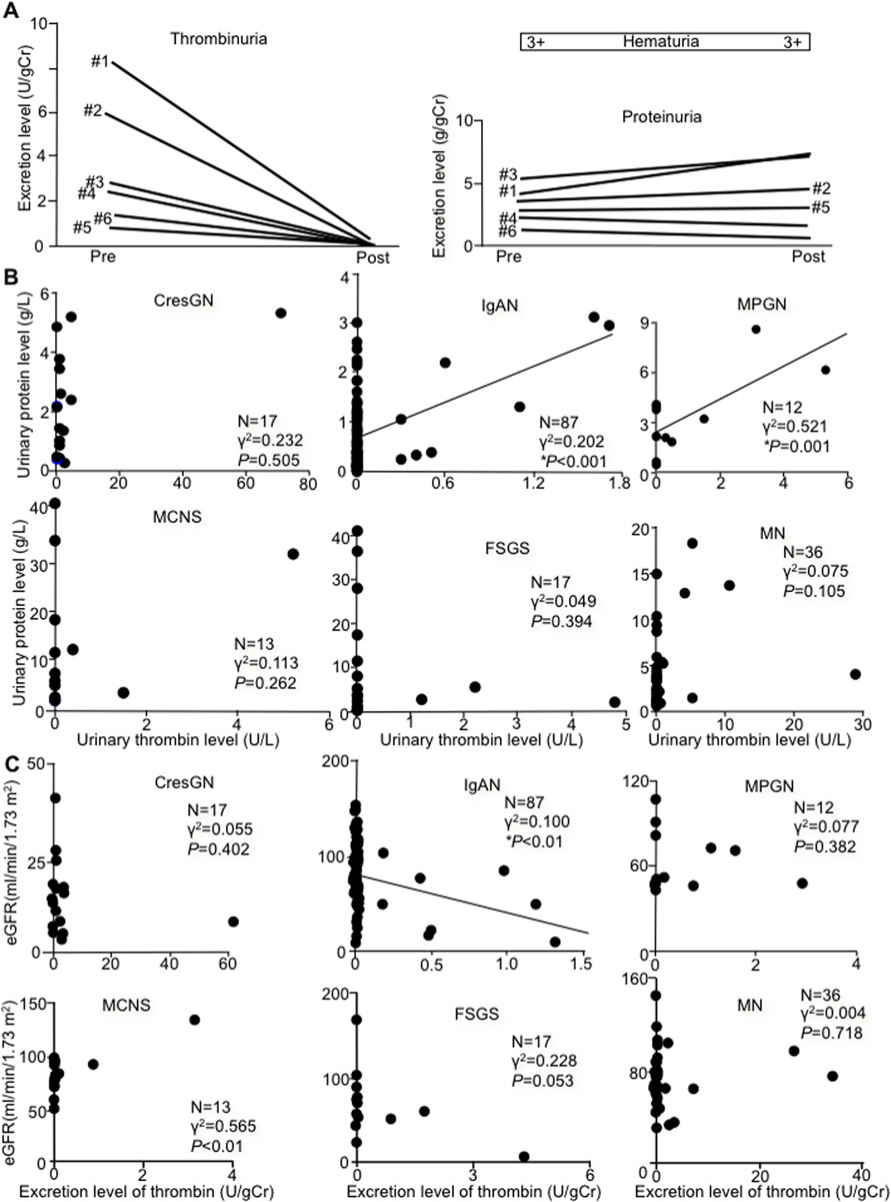
Glucocorticoid effect on thrombinuria and proteinuria and thrombinuria vs. proteinuria or estimated glomerular filtration rate. (**A**) Thrombinuria (left panel) and proteinuria and hematuria (right panel) before (pre) and after (post) steroid pulse treatment in 6 patients with crescentic glomerulonephritis (CresGN) (#1 to #6) with thrombinuria. Three days of pulse therapy with intravenous methylprednisolone at 0.5–1 g/day was followed by oral prednisolone at 20–30 mg/day. gCr indicates gram of urinary creatinine. (**B**) Relation between urinary thrombin and urinary protein levels for various types of glomerulonephritis. Each circle represents an individual patient. (**C**) Relation between excretion level of thrombin and estimated glomerular filtration rate (eGFR) for various types of glomerulonephritis. Each circle represents an individual patient. IgAN, IgA nephropathy; MPGN, membranoproliferative glomerulonephritis; MCNS, minimal change glomerulopathy; FSGS, focal segmental glomerulosclerosis; and MN, membranous glomerulopathy. In (**B**) and (**C**), asterisks (*) and solid lines indicate statistically significant associations and correlation lines, respectively.

After 2 weeks of glucocorticoid treatment, urinary thrombin excretion decreased in all patients, to 4.9% ± 12.7%, on average, of pretreatment values (*P*<0.01). Indeed, all patients with CresGN had undetectable urinary thrombin activity, but proteinuria and hematuria levels remained unchanged (Fig. 3A). MCNS patients had undetectable thrombinuria, and proteinuria decreased by more than 92% (to the normal range in 2 of 3 patients) after treatment. In patients with IgAN, thrombinuria and proteinuria decreased by more than 58% and more than 24% after treatment, respectively.

### Relationship of thrombinuria to proteinuria and eGFR

Proteinuria was significantly associated with thrombinuria in only IgAN and MPGN patients (positive and moderate correlations) (Fig. 3B). No significant difference was observed in proteinuria for thrombinuria-positive and-negative groups in each type of glomerulonephritis. eGFR was associated with thrombinuria in only patients with IgAN (an inverse and weak correlation) (Fig. 3C). IgAN patients with thrombinuria had a lower eGFR than those without thrombinuria (51.2 ± 34.2 versus 84.0 ± 29.1 ml/min/1.73 m^2^ of body surface area, N = 8 and N = 79, respectively) (*P* = 0.004). In the entire cohort (N = 182), thrombinuria was not associated with either proteinuria (*P* = 0.295) or eGFR (*P* = 0.051), as seen in Fig. 3B and C, respectively.

## Discussion

CresGN demonstrated the highest occurrence of thrombinuria and highest thrombin excretion level compared with other glomerulonephritides (Fig. 1A and B); these findings, plus the absence of thrombinuria in healed CresGN, indicate a close and specific link between thrombinuria and CresGN. The presence of thrombinuria in a severe glomerular inflammatory disease such as CresGN [10] and the absence of thrombinuria in healed CresGN, nephrosclerosis (glomerular non-inflammatory disease), TIN (non-glomerular inflammatory renal disease), and healthy volunteers mean that thrombinuria is a pathological sign and probably indicates the existence of inflammation in the glomerulus. In CresGN, monocytes/macrophages infiltrated glomerular extracapillary tissue with fibrin deposits, and these monocytes/macrophages expressed tissue factor (Fig. 2), in agreement with previous reports [11–15], which suggests that monocytes/macrophages in glomerular extracapillary tissues generate thrombin via activating the tissue factor-initiated blood coagulation pathway. This generated thrombin leaks into urine and causes thrombinuria. AGN also features severe glomerular inflammation with leukocyte accumulation but mostly in the glomerular capillary lumen [28], where thrombin is generated by monocytes/macrophages that express tissue factor (Fig. 2A, e); thrombin in circulation, however, is promptly inactivated by antithrombin [29]. Therefore, in AGN, urine contains little active thrombin, and the occurrence of thrombinuria and excretion levels of thrombin in prolifrerative glomerulonephritis follow the order of the intensity of monocyte/macrophage accumulation in glomerular extracapillary tissues (CresGN > MPGN > IgAN), as reported previously [30]. Although monocyte/macrophage infiltration into glomeruli was negligible in MCNS and FSGS, tissue factor expressed in glomerular mesangial areas or on glomerular epithelial cells (Fig. 2A), via cytokine stimulation [31,32], may also induce thrombinuria (Fig. 1A and B). Thus, thrombinuria may specifically indicate glomerular extracapillary inflammation in CresGN, which suggests the possible application of thrombinuria testing for noninvasive diagnosis and screening of CresGN to improve the poor prognosis of this disease. In fact, thrombinuria had high specificity (90.1%) and moderate sensitivity (70.6%) for CresGN diagnosis (Fig. 1C), as seen in the ROC curve analysis of urine measurements in patients with hematuria and proteinuria, which is an essential but nonspecific sign of CresGN.

In Japan, IgAN is the most common glomerulonephritis [33], and no significant differences exist for the ratios of the types of glomerulonephritis between our study (Table 1) and the Japan Renal Biopsy Registry [34], which indicates a lack of bias in the diagnostic selection in our study and supports the reliability of thrombinuria as a novel biomarker for CresGN. When thrombin is generated systemically and released into circulation, thrombin in circulation is promptly inactivated by binding with plasma antithrombin at the heparan sulfate of endothelial cells and forms the TAT [29]. Therefore, the plasma TAT value reflects the amount of thrombin released into circulation from any vessels or tissues. CresGN is a glomerular vasculitis, and pauci-immune CresGN is often associated with systemic vasculitis (ANCA-associated vasculitis [25]), in which thrombin may be released into circulation from vasculitic lesions. Therefore, the lack of association of plasma TAT with urinrary thrombin activity in ANCA-associated CresGN (Fig. 1D) indicates that urinary thrombin activity is not affected by thrombin released into circulation. Inactivation of thrombin during retention in the bladder appears to be negligible, as shown by the results of our *in vitro* experiment, and the lack of association between urinary thrombin and urinary TAT levels (Fig. 1E) suggests insignificant thrombin capture and inactivation by antithrombin (TAT formation) in urine *in vivo*. Thus, the urinary thrombin level is believed to reflect thrombin generation in glomerular tissue. Furthermore, no association of hemoglobinuria (hematuria) with thrombinuria (Fig. 1F), together with persistent hematuria without detectable thrombinuria in CresGN after glucocorticoid treatment (Fig. 3A), indicates that the thrombin generated at glomerular bleeding sites for hemostasis does not significantly affect thrombinuria measurements. Taken together, our data here confirm that thrombinuria is a specific marker of glomerular inflammation and that the thrombin assay is a reliable laboratory test for CresGN diagnosis.

The rapid thrombinuria decrease to undetectable levels after glucocorticoid treatment in CresGN (Fig. 3A), which indicates suppressed glomerular inflammation and tissue factor expression [35], in contrast to persistent proteinuria and hematuria that are caused by a disrupted glomerular structure, suggests an application of thrombinuria for rapid and sensitive evaluation of CresGN inflammation activity.

Glomerular fibrin deposition in biopsy tissue is a coagulation marker used in clinical situations, but this fibrin remains in tissues until plasmin degrades it. In contrast, thrombin in glomerular tissues is supposedly removed from the tissues by continuous urine flow as other glomerular epithelial [36] or mesangial [37] proteins are excreted, so the presence of thrombinuria means the occurrence of blood coagulation in glomerular tissues. The coexistence of thrombinuria and fibrin deposition in half of the patients with CresGN (Table 3) indicates high levels of ongoing activation of blood coagulation in glomerular tissues. However, fibrin deposition and thrombinuria did not coexist in one-third of our patients with CresGN. Fibrin deposition without thrombinuria indicates previous activation of blood coagulation, but thrombinuria without detectable fibrin deposition may be due to a lower sensitivity of the fibrin assay compared with the sensitivity of our thrombin assay. Thus, thrombinuria appears to provide reliable information about active blood coagulation in glomerular tissues at about the time of sampling without a renal biopsy.

ANCA is a marker autoantibody for ANCA-associated vasculitis and is highly positive in patients with pauci-immune CresGN [25] but is still negative in 10–30% of patients with pauci-immune CresGN [6] and in most patients with immune-complex CresGN [7]. As Table 2 shows, 5 (33.3%) of our patients with pauci-immune CresGN had negative ANCA results, but 2 of the 5 patients presented with thrombinuria. Moreover, our 4 patients with pauci-immune CresGN and 2 patients with immune-complex CresGN had positive results for only thrombinuria or ANCAs. Combination of these two independent markers raised the sensitivity for CresGN diagnosis from 70.6% (thrombinuria only) to 82.4% (combination). Thus, combining thrombinuria and ANCA assays may increase the value of thrombinuria for diagnosis and screening of CresGN. Although the following information is not included in Table 1, urinary thrombin activities were measured in 3 patients with proteinase 3-ANCA and in another 3 patients with anti-glomerular basement membrane antibody, all of whom were clinically diagnosed, without a histopathological examination, as having rapidly progressive glomerulonephritis. In 2 of the 3 patients in each group, urinary thrombin activities were present, which may also support the possibility of utilizing thrombinuria for the diagnosis of a broad spectrum of CresGN.

Crescent formation in glomeruli is an essential pathological finding of CresGN, and cellular crescents usually accompany fresh inflammatory changes; however, uneven distribution of crescentic lesions in renal biopsy tissue sections does not always reflect the average crescent formation in whole glomeruli. In contrast, thrombin generated in individual inflammatory glomeruli is collected in the bladder and excreted (thrombinuria), so the thrombin excretion level corresponds to thrombin generation in the whole kidney. Thus, the lack of association between the urinary thrombin excretion level and crescent formation (Table 2) may not affect the reliability of thrombinuria as a novel marker of glomerular inflammation. Inasmuch as thrombinuria is a specific marker of inflammation in glomeruli in CresGN, it showed no correlation with CRP (Table 2) as a systemic inflammation marker, proteinuria as a glomerular injury marker (Fig. 3B), or eGFR as an indicator of glomerular filtration function (Fig. 3C) in CresGN. Significant proteinuria and a reduced glomerular filtraion rate indicate a poor prognosis in IgAN and MPGN [38]. The findings that thrombinuria levels correlated positively with proteinuria levels in IgAN and MPGN (Fig. 3B) and that the eGFR of patients with IgAN and thrombinuria was lower than that of patients without thrombinuria as well as a negative correlation between thrombinuria and eGFR in IgAN (Fig. 3C) suggest that thrombinuria is also a factor indicating a poor prognosis in IgAN and MPGN, particularly IgAN, the most common glomerulonephritis in Japan.

In conclusion, urinary thrombin (thrombinuria) is a novel marker that is specific for glomerular inflammation and is available for noninvasive diagnosis and screening of CresGN. Thrombinuria may also be suitable for evaluation of the activity of various other inflammatory glomerular diseases, and additional studies may be needed to establish the clinical utility of the urinary thrombin test.
